# Maternal control of visceral asymmetry evolution in *Astyanax* cavefish

**DOI:** 10.1038/s41598-021-89702-6

**Published:** 2021-05-13

**Authors:** Li Ma, Mandy Ng, Janet Shi, Aniket V. Gore, Daniel Castranova, Brant M. Weinstein, William R. Jeffery

**Affiliations:** 1 Department of Biology, University of Maryland, College Park, MD 20742 USA; 2 Division of Developmental Biology, Eunice Kennedy Shriver National Institute of Child Health and Human Development, NIH, Bethesda, MD 20892 USA; 3 Present Address: Cave Fish Development and Evolution Research Group, Kunming Institute of Zoology, Chinese Academy of Sciences, Kunming, 650201 Yunnan China

**Keywords:** Developmental biology, Evolution

## Abstract

The direction of visceral organ asymmetry is highly conserved during vertebrate evolution with heart development biased to the left and pancreas and liver development restricted to opposing sides of the midline. Here we show that reversals in visceral organ asymmetry have evolved in *Astyanax mexicanus*, a teleost species with interfertile surface-dwelling (surface fish) and cave-dwelling (cavefish) forms. Visceral organ asymmetry is conventional in surface fish but some cavefish have evolved reversals in heart, liver, and pancreas development. Corresponding changes in the normally left-sided expression of the Nodal-Pitx2/Lefty signaling system are also present in the cavefish lateral plate mesoderm (LPM). The Nodal antagonists *lefty1* (*lft1*) and *lefty2* (*lft2*), which confine Nodal signaling to the left LPM, are expressed in most surface fish, however, *lft2,* but not *lft1,* expression is absent during somitogenesis of most cavefish. Despite this difference, multiple lines of evidence suggested that evolutionary changes in L-R patterning are controlled upstream of Nodal-Pitx2/Lefty signaling. Accordingly, reciprocal hybridization of cavefish and surface fish showed that modifications of heart asymmetry are present in hybrids derived from cavefish mothers but not from surface fish mothers. The results indicate that changes in visceral asymmetry during cavefish evolution are influenced by maternal genetic effects.

## Introduction

Vertebrates are characterized by mirror-image symmetry of external structures and left–right (L-R) asymmetry of many visceral organs^1,2^. L-R asymmetry is first apparent during embryonic development and is important in adult organ packaging, connectivity, and function. During embryogenesis, the cardiac and gut tubes bend asymmetrically, the heart develops with a bias to the left side and endodermal organs are offset to the left or right sides of the midline. The direction of L-R asymmetry is highly conserved^3^. Although reversal of L-R asymmetry can occur in zebrafish and mouse mutants^4,5^, and in about 1 of 10,000 human births^6^, large changes in the conventional mode of visceral asymmetry have not been reported during vertebrate evolution.

The molecular mechanisms of L-R patterning have been extensively studied in traditional vertebrate models^7,8^. An early step in L-R patterning is the leftward beat of cilia in symmetry-breaking organizers, such as the node in mice^9,10^ and Kupffer’s vesicle (KV) in teleosts^11,12^, which transiently form near the posterior end of the notochord during gastrulation. The leftward directional flow is thought to be sensed by lateral organizer cells, which activate the expression of the TGFß-signaling ligand Nodal and the homeobox gene *pitx2* in lateral plate mesoderm (LPM) on the left side of the axis. The Nodal-Pitx2 signaling cascade then spreads from posterior to anterior in the left LPM and initiates an autoregulatory loop involving the TGFß ligands Lefty1 and Lefty2, which confine the asymmetric signal to the left side of the midline by antagonizing Nodal ^13-16^. The stabilized Nodal-Pitx2/Lefty cascade activates downstream regulatory circuits that control organ morphogenesis on the left or right sides of the body axis^17^. Less is known about L-R patterning events upstream of the symmetry-breaking organizer in vertebrates^18,19^, although the initial patterning steps may be controlled by asymmetries in maternal H^+^/K^+^-ATPase mRNA localization and differences in membrane voltage potentials in *Xenopus*^2^. The influence of maternal factors in controlling L-R shell coiling is well known in snails^20,21^

In this study, we examine L-R visceral asymmetry in the teleost *Astyanax mexicanus,* a model system for studying the evolution of development consisting of a surface-dwelling form (surface fish) and multiple cave-dwelling (cavefish) forms^22^. *S*urface fish and cavefish evolved from a common surface-dwelling ancestor about 20,000–200,000 years ago^23,24^. Cavefish have evolved novel traits as a response to the challenging cave environment^25^. Although the most famous cavefish traits are the loss of eyes and pigmentation, cavefish also gain many traits, such as unusual L-R asymmetry of the jaws, skull shape, and the distribution of cranial neuromasts, which may have evolved to assist in feeding or navigation in darkness^26–28^. The known changes in cavefish L-R patterning are initiated during later development^28^, and little attention has been given to the possibility of asymmetric differences between surface fish and cavefish embryos.

We show here that surface fish embryos exhibit conventional L-R asymmetry of the Nodal-Pitx2/Lefty signaling cascade, heart, liver, and pancreas but cavefish have evolved significant changes of these asymmetries. The results of reciprocal hybridization experiments, the fertilization of cavefish eggs with surface fish sperm and vice versa, reveal that these evolutionary changes in cavefish L-R asymmetry are influenced by maternal genetic effects.

## Results

### Changes in cavefish visceral asymmetry

**Figure 1:**
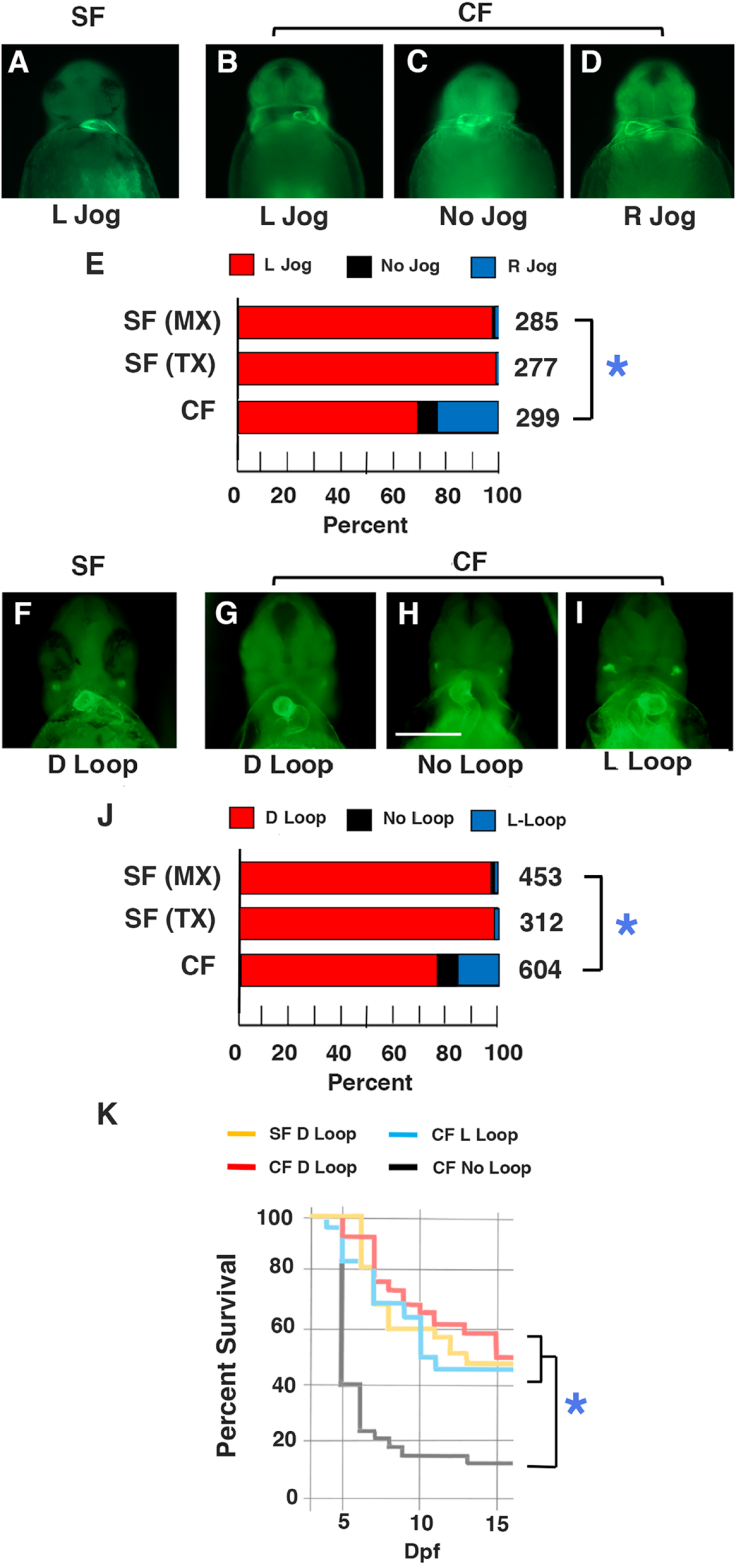
Changes in heart asymmetry in cavefish. (**A**–**E**) Heart jogging. (**A**) Surface fish (SF) with left-jogging heart tube. Cavefish (CF) with left-jogging (**B**), non-jogging (**C**), or right-jogging (**D**) hearts. (**E**) Bar graphs showing the proportion of left-jogged, non-jogged, and right-jogged cardiac tubes in Mexican surface fish (SF-MX), Texas surface fish (SF-TX), and cavefish (CF) at 1.5 dpf. The numbers of assayed fish are shown at the right of each bar. Asterisk: Chi^2^ statistic = 180.743; *p* < .00001. (**F**–**J**) Heart looping. (**F**) Surface fish with D-looping hearts. (**G**–**I**) Cavefish with D-looping (**G**), non-looping (**H**), and L-looping (**I**) hearts. (**J**) Bar graphs showing the proportion of D-looping, non-looping, and L-looping hearts in Mexican surface fish (SF-MX), Texas surface fish (SF-TX), and cavefish (CF). The number of assayed fish is shown at the right of each bar. Asterisk: Chi^2^ statistic = 145.907; *p* < .00001. Larvae were stained with MF-20 antibody and viewed from the ventral side at 1.5 dpf for heart jogging or at 3.5 dpf for heart looping. Scale bar in H: 250 µM; magnification is the same in all frames. (**K**) Survival of cavefish with differences in heart laterality. Graph shows the percentage of surviving surface fish of an initial 100 larvae with D-looped hearts and cavefish of an initial 75 larvae with D-looped hearts, an initial 38 larvae with non-looped hearts, and an initial 52 larvae with L-looped hearts on each day beginning at 3 dpf. Red, blue, and yellow survival profiles show no significant differences. Black survival profile shows a significant difference (*p* < .000) compared to all other groups.

The polarity of heart, liver, and pancreas development were compared in surface fish and cavefish. The heart primordium is the first organ precursor to show L-R asymmetry in vertebrate embryos^29^. The cardiac tube forms along the ventral midline, then jogs to the left (left-jogging) and the jogged heart later loops to the right (D-looping), giving rise to an S-shaped organ. The direction of cardiac jogging was determined by staining larvae with myosin heavy chain antibody M-20^30,31^ at about 1.5 days post-fertilization (dpf) (Fig. 1A–E). More than 98% of the larvae from surface fish populations originating in Texas and Mexico showed left-jogging cardiac tubes (Fig. 1A,E). In contrast, only 68% of cavefish larvae had left-jogging cardiac tubes (Fig. 1B,E), and the remainder showed either right-jogging (Fig. 1D,E) or non-jogging cardiac tubes (Fig. 1C,E). Myosin heavy chain staining was also used to determine the direction of heart looping in surface and cavefish larvae at 3 dpf (Fig. 1F–J). More than 97% of Texas and Mexican surface fish larvae showed D-looping hearts (Fig. 1F,J), whereas about 76% of cavefish larvae had D-looping hearts (Fig. 1G,J), 17% showed left-looping (L-looping) hearts (Video; Fig. 1I,J), and 7% had non-looping hearts (Fig. 1H,J). Heart looping was consistent in different surface fish families but varied considerably among different cavefish families: some cavefish families (e.g. PA61) exhibited almost 40% abnormal heart looping, some (e.g. PA72) exhibited more moderate levels of altered heart looping, and others showed mostly D-looping hearts (Fig. S1). We also followed heart looping in single clutches of surface fish and cavefish larvae into later stages of development, and the results showed that the same proportions of normal (surface fish) or abnormal (cavefish) looping were present at 3, 6, 9 and 19 dpf (Fig. S2). These results indicate that the direction of L-R heart asymmetry is reversed in a significant proportion of cavefish.

**Figure 2:**
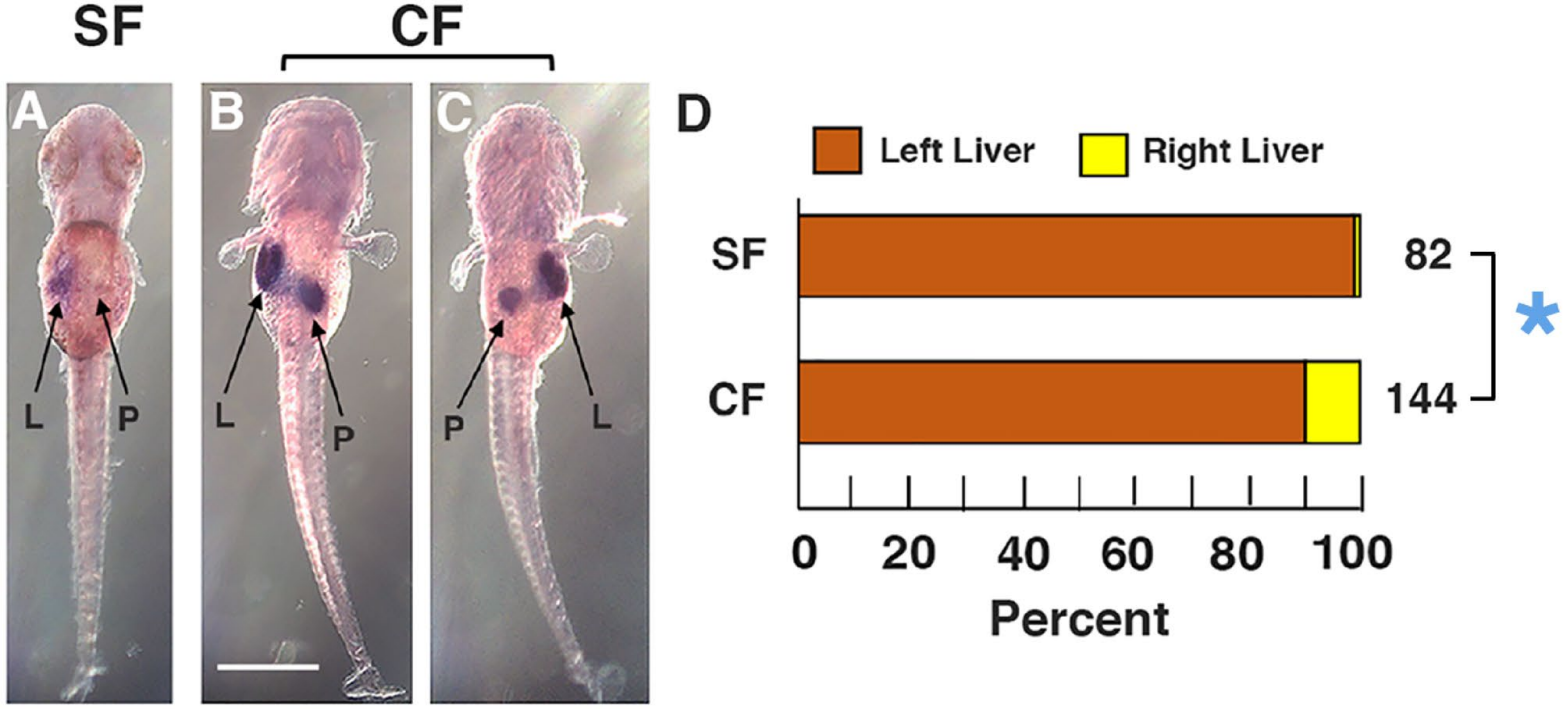
Liver and pancreas laterality in surface fish and cavefish determined by *cbsa* gene expression. (**A**–**C**) In situ hybridization showing *cbsa* expression in liver positioned on the left and pancreas positioned on the right of the midline in surface fish (SF) (**A**), and normal (**B**) and reversed (**C**) liver-pancreas laterality in cavefish (CF) at 3.5 dpf. All views from the dorsal side. L: liver. P: pancreas. Scale bar in B is 500 µm; magnification is the same in all frames. (**D**) Bar graphs showing the percentage of livers positioned on the left or right side in surface fish and cavefish. Total number of in situ hybridized larvae is shown at the right of each bar. Asterisk: Chi^2^ statistic = 3.6603, *p* = .055723.

We next compared the pattern of L-R asymmetry in the liver and pancreas of surface fish and cavefish larvae. These organs form as tubular protrusions of the gut, which subsequently shift from the midline and continue to develop on the left or right sides of the body respectively^32^. The positions of liver and pancreas development were compared in 3.5 dpf surface fish and cavefish larvae by in situ hybridization using the *cystathionine ß-synthase a* (*cbsa*) gene (Fig. 2), which is strongly expressed in these organs during *A*.* mexicanus* development^33^. Consistent with conventional L-R laterality in zebrafish^35^, the liver developed on the left and the pancreas on the right in more than 98% of surface fish larvae (Fig. 2A,D). However, only about 89% of cavefish larvae showed the typical L-R relationship of these organs, while in the remaining 11% the liver developed on the right and the pancreas on the left of the midline (Fig. 2B–D). To determine whether the reversal in liver and pancreas positioning were complementary to the heart reversals, we examined cardiac looping in *cbsa* stained cavefish embryos. The results indicated that most cavefish embryos with normal left-sided liver primordia showed corresponding normal heart D-looping, and embryos with abnormal right livers showed corresponding abnormal heart L-looping (Fig. S3). However, there was also a small subset of embryos with left or right sided livers that showed L- or D-looping hearts, respectively (Fig. S3). Therefore, most cavefish exhibit corresponding reversals of liver and heart asymmetry (*situs inversus totalis*^6^), but in a few cases reversals in these organs can occur independently (heterotaxy^6^). We conclude that cavefish have evolved changes in L-R asymmetry of the heart, liver, and pancreas.

To determine the effects of changes in cavefish L-R heart asymmetry on viability, we compared the survival of cavefish larvae with different heart looping asymmetries (Fig. 1K). At 3 dpf, cavefish larvae were separated into groups with D-looped, non-looped, and L-looped hearts by visual inspection (see Video), and the number of living larvae in each group, as well as in surface fish controls, was followed for the next 14 days. The surface fish larvae used in this experiment, all with D-looped hearts, showed about 50% survival, which is typical for our culture conditions^33^. As described above, the cavefish larvae used in this experiment showed modifications in conventional L-R asymmetry: about 45% had D-looping hearts, 32% had L-looping hearts, and 23% had hearts without looping. Similar to the human condition *situs inversus totalis*^6^, no significant differences were seen between the survival of surface fish larvae and cavefish larvae with D-looped or L-looped hearts (Fig. 1K). In contrast, survival was significantly lower in cavefish larvae without heart looping, and most of these larvae gradually developed heart cavity edemas and perished (Fig. 1K). The results suggest that complete reversal of heart looping does not affect cavefish survival, at least during the larval stages, but in most cases the absence of cardiac looping is lethal.

### Changes in nodal-pitx2/lefty expression in cavefish

**Figure 3:**
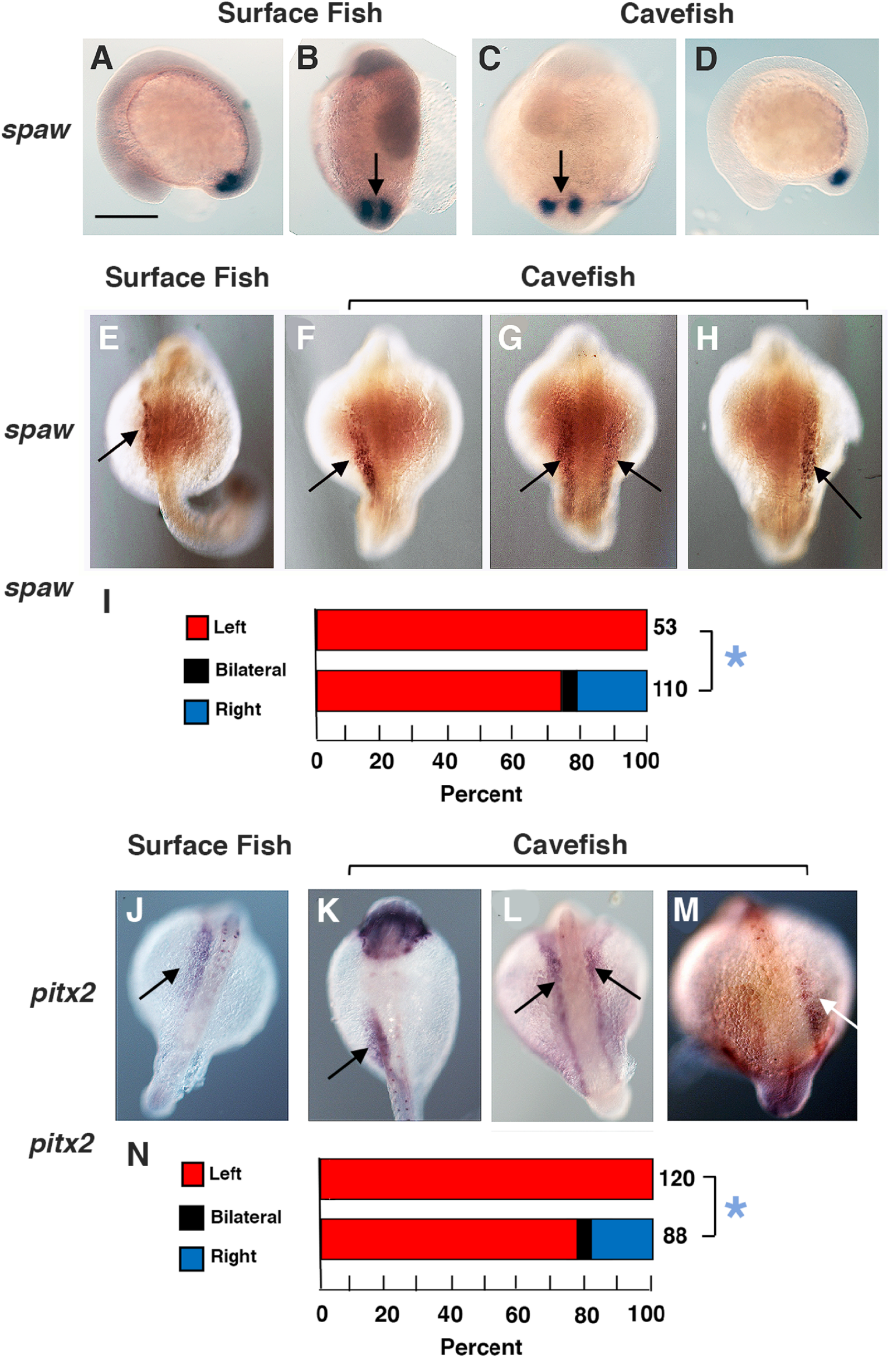
Changes in Nodal-Pitx2 expression in cavefish embryos during segmentation. (**A**–**H**) In situ hybridizations showing *spaw* expression around Kupffer’s vesicle (arrows in **B**, **C**) and in the lateral plate mesoderm (LPM, arrows in **E**–**H**) at the 10–13 somite (**A**–**D**) and 18–25 somite (**E**–**H**) stages in surface fish (**A**, **B**, **E**) and cavefish (**C**, **D**; **F**–**H**) embryos. (**J**–**M**). In situ hybridizations showing *pitx2* expression in the LPM (arrows) of 25–30 somite surface fish (**J**) and cavefish (**K**–**M**) embryos. (**A**, **D**) lateral views with anterior to the left. (**B**, **C**, **E**–**H**, **J**–**M**) Dorsal views with anterior on the top. Scale bar in (**A**): 100 µm; magnification is the same in all frames. (**I**, **N**) Bar graphs showing number of embryos with *spaw* (**I**) or *pitx2* (**N**) expression in the left, left and right, and right LPM in surface fish and cavefish embryos. The numbers of embryos analyzed are shown at the right of each bar. Asterisk in I: Chi^2^ statistic = 11.5372, *p* = .003124. Asterisk in H: Chi^2^ statistic = 24.1241, *p* < .00001.

Nodal expression begins in bilateral domains surrounding the KV and later continues in the left LPM, where *pitx2* expression is activated, while signaling is suppressed in the right LPM^36^. To determine if changes in cavefish visceral organ laterality are associated with alterations in the L-R asymmetry of Nodal/Pitx2 signaling, expression of the teleost *nodal* paralog *southpaw* (*spaw)*^36^ and *pitx2* were compared in segmenting surface fish and cavefish embryos by in situ hybridization. At the 8–10 somite stage, *spaw* was expressed bilaterally around the KV in both surface fish and cavefish embryos (Fig. 3A–D), implying that Nodal signaling begins normally on both sides of the L-R axis during the early segmentation stages. During the 18–25 somite stages, surface fish embryos expressed *spaw* and *pitx2* in the left LPM, but not in the right LPM (Fig. 3E,I,J,N), indicating the Nodal-Pitx2 system was expressed normally. In contrast, *spaw* and *pitx2* were expressed in the left LPM in only about 75% of cavefish larvae, whereas these genes were expressed in the right LPM in about 20% and bilaterally in about 5% of the cavefish embryos (Fig. 3F–I,K–N), approximately the proportion showing abnormal heart tube looping (Fig. 1J). These results indicate that the left-sided asymmetry of Nodal-Pitx2 expression is modified in cavefish.

**Figure 4:**
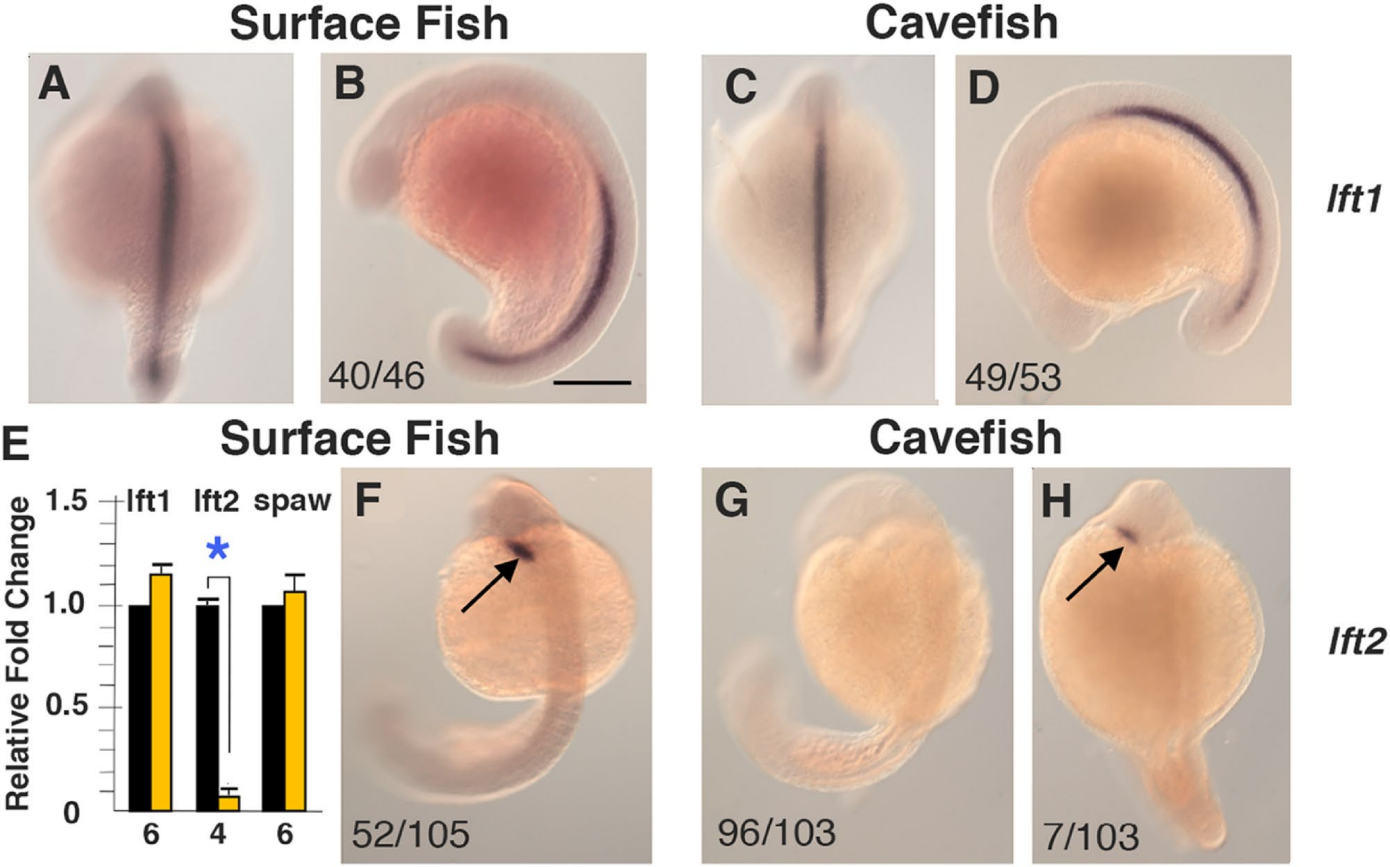
The expression of *lefty* genes in surface fish and cavefish embryos during segmentation. (**A**–**D**) In situ hybridization showing *lft1* expression along the dorsal midline in 10–13 somite surface fish (**A**, **B**) and cavefish (**C**, **D**) embryos. (**A**, **C**) Dorsal views with anterior on the top. (**B**, **D**) Lateral views with anterior on the left. (**F**–**H**) In situ hybridization showing *lft2* expression in the LPM (arrows) in 18–25 somite surface fish (**F**) and cavefish embryos (**G**, H). Dorsal views. The numbers in the frames indicate embryos with the indicated expression pattern compared to the total number of in situ hybridized embryos. Scale bar in (**B**): 100 µm; magnification is the same (**A**–**D**) and (**F**–**H**). (**E**) Bar graph showing relative fold changes in *lft1*, *lft2*, and *spaw* mRNA levels in cavefish compared to surface fish determined by qPCR at the 18–25 somite stage. Black bars: surface fish. Yellow bars: cavefish. Error bars: SEM. Number of replicates shown at the bottom of the columns. Asterisk: *p* < .01.

We next asked what molecular changes control the modifications in Nodal-Pitx2 signaling in cavefish. The Nodal-Pitx2 cascade is restricted to the left LPM by Lefty1 and Lefty2, which antagonize Nodal and prevent its diffusion or ectopic expression in the right LPM^13–16^. To determine whether the Lefty antagonists are expressed normally, we compared *lft1* and *lft2* expression in surface fish and cavefish embryos during the segmentation stages. In situ hybridization showed that *lft1* is expressed along the midline in both 18–23 somite surface fish and cavefish embryos (Fig. 4A–D), implying that the Lefty1 barrier persists in cavefish. In contrast, although *lft2* is expressed normally in the anterior left LPM in about 50% of the surface fish embryos at the 25-somite stage, *lft2* expression could not be detected in either the left or right LPM in the majority of 25-somite cavefish embryos (Fig. 4F–H). The *lft2* gene was expressed weakly in about 6% of 18-somite cavefish embryos, but only in the left LPM (Fig. 4G,H). We conducted several additional studies to substantiate these results. First, the cavefish embryos were subjected to in situ hybridization to detect both *lft1* and *lft2* in the same embryos, and all cavefish embryos stained positive for *lft1* while none showed *lft2* staining (Fig. S4). Second, in situ hybridization was conducted on surface fish and cavefish embryos distributed from 12 h of development through the 25-somite stage, and *lft2* staining was also absent in the cavefish LPM at stages of segmentation earlier and later than the 18-somite stage (Fig. S5). Lastly, to confirm *lft2* downregulation and test the consequences on *spaw* expression, we quantified the levels of *lft1*, *lft2*, and *spaw* mRNA by qPCR during the 18–25-somite stages (Fig. 4E). No significant changes were seen in *lft1* or *spaw* mRNA levels between surface fish and cavefish, but a significant decrease in *lft2* occurred in cavefish relative to surface fish embryos. The results indicate that *lft2* expression is downregulated in the cavefish LPM during the segmentation stages, opening the possibility that this change may be involved in modifying L-R asymmetry.

### Role of lefty2 in cavefish L-R patterning

Several different experiments were conducted to further investigate the basis for defective *lft2* expression and its possible role in the evolution of L-R visceral asymmetry in cavefish.

**Figure 5:**
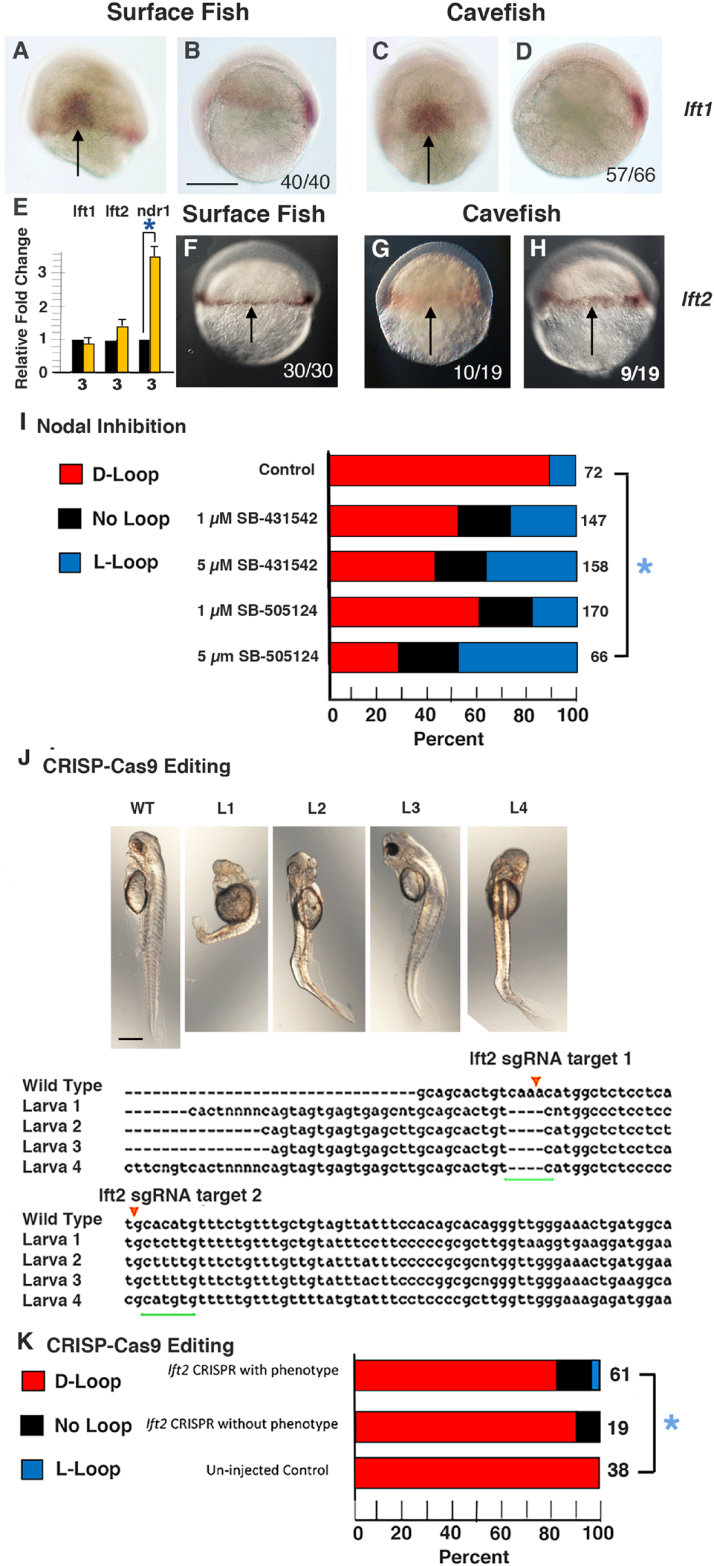
Role of *lefty2* downregulation in Nodal/Pitx2 signaling and heart L-R asymmetry. (**A**–**D**, **F**–**H**) In situ hybridization showing *lft1* (**A**–**D**) and *lft2* (**F**–**H**) expression in surface fish (**A**, **B**, **F**) and cavefish (**C**, **D**, **G**, **H**) at 50% epiboly. (**A**, **C**) Dorsal views. (**B**, **D**, **F**–**H**) Lateral views. Arrows in (**A**, **C**) indicate the shield region and in (**F**–**H**) indicate the germ ring. The numbers in the frames indicate embryos with the indicated expression pattern compared to the total number of in situ hybridized embryos. Scale bar in B: 150 µm; magnification is the same in all frames. (**E**) Bar graphs showing relative fold changes in *lft1*, *lft2*, and *ndr1* mRNA in cavefish compared to surface fish gastrulae determined by qPCR at the 50% epiboly stage. Black bars: surface fish. Yellow bars: cavefish. Error bars: SEM. Number of replicates shown at the bottom of the columns. Asterisk: *p* < 0.01. (**I**) Effects of the Nodal inhibitors SB-431542 and SB-505124 on heart L-R asymmetry in cavefish. Bar graphs showing the proportion of D-looped, non-looped, and L-looped hearts in cavefish controls and 1 µM and 5 µM SB431542 or SB-505124 treated larvae stained with MF-20 antibody at 3 days post-fertilization (dpf). The numbers of assayed fish are shown at the right of each bar. Asterisk: Chi^2^ statistic = 62.2813; *p* < 0.00001. (**J**, **K**) Effects of *lft2* CRISPR-Cas9 on heart looping in surface fish at 3 dpf. (**J**) Examples of larvae (**L**) with axial defects (L1–4, top) and mutated *lft2* sequences (bottom, underlined in green) in 4 injected surface fish compared to a wild type surface fish control (WT) from the same clutch. Red arrows: sgRNA target. Scale bar is 100 µm; magnification is the same in all frames. (**K**) Bar graphs showing proportion of heart looping asymmetry in genotyped injected surface fish with (top) or without (middle) mild axial phenotypes compared to controls from the same clutch (bottom). The numbers of assayed fish are shown at the right of each bar. Asterisk: Chi^2^ statistic = 6.1667; *p* = .187043.

First, we asked whether *lft2* downregulation is restricted to the LPM or is more general during cavefish development. The *lft2* gene is also expressed during gastrulation^37,38^; thus, *lft2*, and as a control *lft1*, expression was compared in surface fish and cavefish at about 50% epiboly (Fig. 5A–H). In situ hybridization indicated that *lft1* is expressed in the shield region of both surface fish and cavefish gastrulae (Fig. 5A–D), as described previously^39^, and *lft2* is expressed in the germ ring in surface fish (Fig. 5F) and cavefish gastrulae, although *lft2* staining levels varied in individual cavefish embryos (Fig. 5G,H). The in situ hybridization results were confirmed by qPCR, which indicated no significant differences in *lft2* or *lft1* mRNA levels between surface fish and cavefish gastrulae, although higher levels of *nodal related 1* (*ndr1*) mRNA were detected in cavefish (Fig. 5E), as described by others^40^. These results suggest that *lft2* downregulation is restricted to the LPM during cavefish segmentation.

Second, to explore the possibility of coding mutations in *lft2*, the four *lft2* exons were amplified and sequenced in genomic DNA from individual male and female cavefish whose offspring showed high levels of reversed heart asymmetry (Fig. S6). No differences were uncovered in the nucleotide sequences of the *lft2* exons in cavefish of either sex compared to the surface fish *lft2* gene (Fig. S6), indicating that the *lft2* coding region is intact. Therefore, downregulation of *lft2* expression is probably controlled by change in non-coding regulatory regions.

Third, additional experiments were performed to test the role of *lft2* in heart L-R asymmetry. In one of these, the effects of the small molecule drugs SB-431542 and SB-505124 on heart looping asymmetry were examined in cavefish. These drugs inhibit Nodal-Pitx2 signaling by interfering with Nodal receptor function^39–42^. If cavefish heart L-looping is caused by increased Nodal/Pitx2 activity due to relaxed antagonism by Lefty2, then Nodal inhibition would be expected to promote an increased proportions of D-looping in cavefish. Instead, we found that SB-431542 or SB-505124 treatment decreased the proportion of D-looping in drug treated embryos relative to controls (Fig. 5I), which is inconsistent with relaxed Lefty2 antagonism of Nodal as the cause of reversed heart asymmetry in cavefish. In other experiments, we investigated the effects of CRISPR-Cas9 editing the *lft2* gene on heart looping asymmetry in surface fish. Surface fish eggs were injected with CRISPR-Cas9 and 2 sgRNAs, and some of the larvae with visible axial defects were selected for genotyping (Fig. 5J). The remaining larvae were separated into groups with and without axial effects, and both groups were assayed for heart looping by myosin heavy chain staining. Although the proportion of embryos without heart looping was increased in the CRISPR-Cas9 edited embryos, only small increases in L-looping hearts occurred in both groups of CRISPR-Cas9 injected larvae, which were statistically insignificant when compared to wild-type controls from the same clutch (Fig. 5K). These results suggest that CRISPR-Cas9 mediated *lft2* mutagenesis does not have a major effect on conventional heart looping asymmetry.

In summary, these experiments imply that despite *lft2* downregulation, evolutionary changes in cavefish heart L-R asymmetry are unlikely to be caused by reduced Lefty2 antagonism of Nodal-Pitx2 signaling.

### Cavefish heart looping asymmetry is influenced by maternal genetic effects

**Figure 6:**
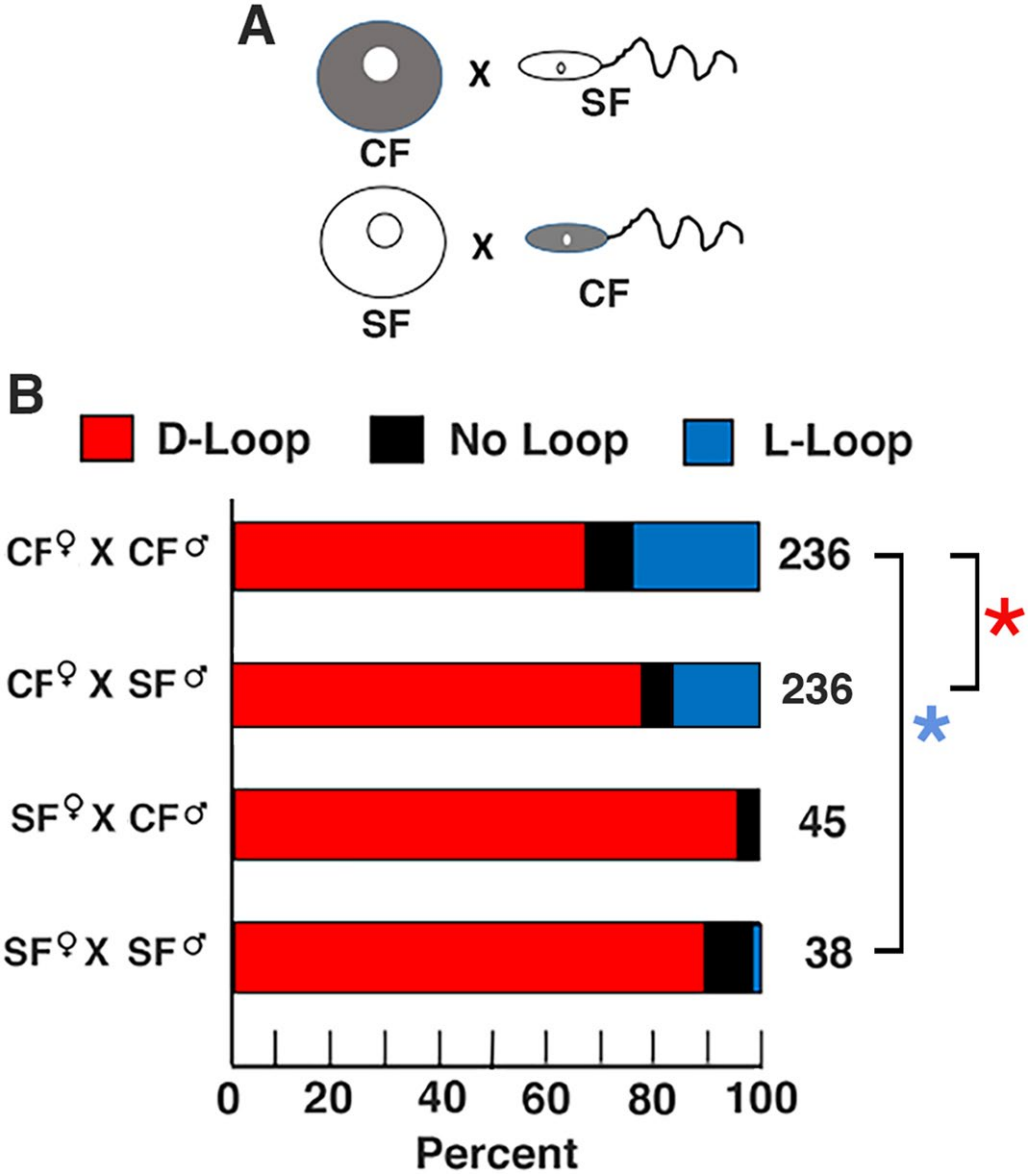
The influence of maternal genetic effects on heart L-R asymmetry. (**A**) Reciprocal crosses between surface fish (SF) and cavefish (CF) gametes in both directions were used to assay for maternal genetic effects. (**B**) Bar graphs showing the percentage of heart looping types in the F1 progeny of CF × CF (top row), CF female × SF male (second from top row), SF female × CF male (second from bottom row), and SF × SF (bottom row) crosses. The number of larvae analyzed are indicated at the right of each bar. Blue asterisk: Chi^2^ statistic (all crosses) = 26.7158; *p* = .000164. Red asterisk. Chi^2^ statistic (CF × CF and CF × SF crosses) = 7.007; *p* = .030091.

Because the results described above did not support a role for Nodal antagonism in changing cavefish L-R asymmetry, we next investigated the possibility that cavefish L-R patterning is controlled at an earlier developmental stage. Previous studies have shown that some cavefish traits are under maternal genetic control^39,43^. To distinguish between maternal and zygotic effects, we conducted reciprocal hybridizations, fertilization of cavefish eggs with surface fish sperm and fertilization of surface fish eggs with cavefish sperm, and compared the proportion of heart looping asymmetry in the hybrid progeny by myosin heavy chain staining (Fig. 6A). As controls, the same surface fish used in the reciprocal hybridizations were crossed with surface fish, the same cavefish used in reciprocal hybridizations were crossed with cavefish, and the progeny were assayed for heart looping asymmetry. The results showed that heart asymmetry changes in the hybrids were dependent on the source of the eggs: cavefish (female) X surface (male) hybrids showed heart laterality changes similar to cavefish X cavefish controls, including significant levels of L-looped, non-looped, and D-looped hearts, whereas surface fish (female) X cavefish (male) hybrids showed high prevalence of D-looped hearts resembling the surface fish X surface fish progeny (Fig. 6B). However, the proportion of cavefish X surface fish hybrids with L-looping hearts did not reach the level seen in the cavefish X cavefish controls, and this result was significant (Fig. 6B). The results suggest that the evolutionary changes in cavefish L-R heart asymmetry are affected by both maternal and zygotic processes but are strongly influenced by maternal genetic effects.

## Discussion

The position of visceral organs on the left or right side of the body is conserved in all vertebrate groups in which it has been studied^1–4^. In *A*.* mexicanus* surface fish, heart morphogenesis begins on the left side of the midline, initiated by left-jogging and D-looping cardiac primordia, and the liver and pancreas develop on the left and right sides of the midline respectively. The same high predominance of D-looping hearts was found in *A*.* mexicanus* surface fish populations located in the far northern extent of the species range in Texas and the southern extent of the range in Mexico, suggesting that this is the ancestral and conserved mode of heart asymmetry in *A*.* mexicanus*. In striking contrast, the conventional L-R patterning of visceral organs is changed in *A*.* mexicanus* cavefish embryos, which show a significant proportion of hearts with right-jogging and L-looping, and reversal in the normal polarity of liver and pancreas development. Changes in cavefish heart polarity persist during larval and fry development, at least up to 19 dpf, and possibly in adults (see below). In most cases, corresponding reversals were noted between heart and liver/pancreas asymmetry, suggestive of *situs inversus totalis*^6^. However, there was also evidence of independent reversals of asymmetry in the heart and liver/pancreas, suggesting heterotaxy^6^. Therefore, cavefish appear to have evolved both complete and incomplete reversals of visceral organ asymmetry. The complete reversal of L-R heart asymmetry had no effects on survival, which is similar to the development of healthy humans exhibiting *situs inversus totalis*^6^, and also explains the inheritance of this phenotype in cavefish. However, the absence of heart looping appeared to be lethal. Consistent with changes in visceral organ L-R asymmetry, the Nodal-Pitx2 signaling cascade, which controls the L-R patterning of visceral organs^7,8^, can be expressed in the left or right LPM or bilaterally in cavefish, rather than only in the left LPM as in surface fish and other vertebrate embryos. These results indicate that cavefish visceral asymmetry is controlled by alterations in Nodal/Pitx2 signaling.

The differences in L-R heart asymmetry are interesting in light of other changes that have recently been noted between cavefish and surface fish hearts. According to Tang et al.^44^, the cavefish heart is smaller and the ventricle more rounded than its surface fish counterpart. These morphological changes appear early in development, about the same time that cardiac jogging and looping are offset from the midline. The cavefish heart also appears to beat more slowly than the surface fish heart^44^. Furthermore, while surface fish hearts are capable of regeneration after injury, as in other teleosts^45^, cavefish hearts cannot be completely replaced and remain permanently scarred^46^. Whether these morphological and physiological changes in cavefish hearts are related to the reversal of asymmetry is unclear, but the path to investigating this possibility is now open.

Our results suggest that cavefish may be the first example of a vertebrate species showing evolutionary changes in the direction of visceral organ asymmetry. A very small proportion of surface fish with L-looping hearts (> 2%) was present within a large predominance of surface fish with conventional D-looping hearts. This suggests that standing genetic variation exists in *A*.* mexicanus* surface fish populations to account for the evolution of L-R asymmetry reversal in the derived cavefish populations. Along a similar line, different cavefish, but not surface fish, families varied significantly in the proportion of L-looping hearts. The reason for this variation is unclear but it could be based on incomplete fixation of the cavefish allele(s) responsible for modified L-R determination.

The evolution of L-R patterning changes in cavefish could occur by the accumulation of neutral mutations^47^, possibly boosted by population bottlenecks during cave colonization or subsequent emigration underground, or through positive selection^48^, although the direct benefits of visceral asymmetry reversal are not obvious. Another intriguing possibility is that evolutionary changes in L-R asymmetry could be driven by indirect selection related to energy conservation in the resource depleted cave environment^49^. This could occur if L-R symmetry determination is costly, for example because of energy expenditure during ciliary beating in the KV, unconstrained, and free to randomize in the cave environment. It is uncertain whether the changes in molecular or organ asymmetry discovered in cavefish are related to the subsequent development of asymmetry in adult cranial features^26–28^, but if so, then any benefits of shifting L-R pattern could be linked to those of cranial asymmetry.

The reversal of Nodal-Pitx2 signaling from the left LPM to the right LPM or its bilateral activity in both LPMs prompted an investigation of the Lefty feedback system in cavefish. Although the midline barrier to Nodal expansion based on expression of the *lft1* gene is likely to be intact in cavefish, the barrier defined by *lft2* expression in other vertebrates, which normally functions in the heart primordial region of the left LPM, was absent in most cavefish embryos. Although the virtual absence of *lft2* expression in the cavefish LPM was a robust finding, several lines of evidence do not support a role for *lft2* downregulation in the evolution of cavefish L-R asymmetry. First, *lft2* expression was also missing in about 50% of surface fish embryos, nevertheless more than 97–98% showed the conventional L-R heart asymmetry. Furthermore, the proportion of cavefish embryos lacking *lft2* expression in the LPM was much higher than the number showing abnormal heart asymmetry. Second, Nodal inhibition, which according to the hypothesis for abnormal visceral asymmetry based on relaxed Lefty2 antagonism would have been expected to shift cavefish from L- to D-looping hearts, instead caused a decrease in D-looping hearts. Third, CRISPR-Cas9 mutation of the *lft2* gene did not significantly affect the pattern of L-R heart asymmetry in surface fish. Supporting the latter finding, genetic or molecular ablation of *lft2* also has minimal effects on L-R asymmetry in zebrafish embryos^38,50^, where it has been concluded that Nodal signaling in the absence of Lefty2 feedback is still functional. It is also worth mentioning that the *lft2* gene is missing from the genomes of some teleosts (fugu and 2 flounder species) without consequences on L-R patterning^51^. In these species, Lefty1 is presumably sufficient to maintain Nodal-Pitx2 in the left LPM. Lastly, in zebrafish, loss of the *lft2* barrier results in increased *spaw* expression that “loops” anteriorly across the midline and then becomes expressed in the right LPM^16^, but increased expression or anterior looping of *spaw* was not seen in cavefish. Together, these results suggest that downregulation of cavefish *lft2* gene expression may not be a major cause of changes in visceral organ asymmetry.

The lack of evidence for changes at the level of Nodal antagonism in cavefish re-directed our attention to alternatives based on changes upstream of the Nodal-Pitx2/Lefty signaling system. Accordingly, reciprocal hybridization, in which heart looping was compared in the hybrid progeny of cavefish eggs fertilized by surface fish sperm and surface fish eggs fertilized by cavefish sperm^43^, indicated that the proportion of L-looping hearts is strongly influenced by the source of eggs. Most hybrids derived from surface fish eggs show conventional D looping hearts, while a significant proportion of hybrids derived from cavefish eggs show the heart L-looping phenotype characteristic of cavefish. The maternal component(s) present in cavefish eggs that are responsible for downstream changes in visceral asymmetry are currently unknown. It is noteworthy, however, that recent transcriptome analysis indicates the existence of considerable transcript divergence between cavefish and surface fish 2-cell embryos^39^. Furthermore, enzymes and channel proteins of maternal origin have polarized left–right distributions during early cleavages in *Xenopus*^2^. Future studies on these maternal proteins in cleaving *Astyanax* embryos may offer further directions for investigating the maternal contribution to left–right visceral asymmetry. In our reciprocal hybridization experiments, the proportion cavefish x surface fish hybrids with L-looping hearts did not reach the levels of cavefish, suggesting that that there may also be some zygotic influences. The zygotic processes may function between the cleavage stages and KV formation, which could offer another future target for analysis of the developmental mechanisms of evolutionary change in cavefish L-R patterning.

The reciprocal hybridization experiments described in the present investigation support a predominantly maternal origin for modifications in L-R asymmetry during cavefish evolution. Therefore, *A*.* mexicanus* may be an excellent model for determining the molecular mechanisms responsible for the first L-R symmetry-breaking events during vertebrate development.

## Methods

### Animal husbandry and biological procedures

Laboratory raised *A*.* mexicanus* were descendants of original surface fish collected at Nacimiento del Rio Choy, San Luis Potosi, Mexico (Mexican surface fish) and at San Solomon Springs in Balmorhea,Texas (Texas surface fish), and of original cavefish collected at La Cueva de El Pachón, Tamaulipas, Mexico. Families consisting of 10–20 individual male and female siblings of third generation progeny of wild captured surface fish and cavefish were raised under identical conditions at 22–23 °C in a constant water flow system, fed a diet of TetraMin Pro flakes (Tetra Holding Inc, Blacksburg VA) and black worms (Eastern Aquatics, Lancaster, PA), and induced to spawn by excess feeding and gradual increase of water temperature to 25–26 °C^52^. Reciprocal hybridization of cavefish females X surface fish males and surface fish females X cavefish males was carried out by in vitro fertilization or paired mating as described previously^43^. Embryos and larvae were cultured at 23 °C and fed living brine shrimp beginning at about 6 days post-fertilization (dpf).

### Approval for animal experiments

Experimental protocols were conducted in accordance with approved guidelines of the University of Maryland, College Park (IACUC #R-NOV-18-59), the experimental protocols were approved by the University of Maryland animal welfare committee (Project 1241065-1), and the study was carried out in compliance with ARRIVE guidelines.

### Video production

Cavefish larvae at 3 dpf were mounted in 1.5% low melting point agarose dissolved in embryo culture medium^53^. Videos were acquired using a Leica M205 stereo microscope with a Lecia DFC 7000 camera.

### Determination of cardiac asymmetry

The direction of cardiac tube jogging and looping was determined at 1.5 dpf and 3–3.5 dpf respectively by staining with the myosin-heavy chain antibody MF-20^30,31^ (Developmental Studies Hybridoma Bank, University Iowa, Iowa City, IA). Larvae were fixed with 4% paraformaldehyde (PFA) in Phosphate Buffered Saline (PBS) at 4 °C overnight, washed three times with PBST (PBS, 0.5% Triton X-100), dehydrated with an increasing methanol series (25%, 50%, 75%) to 100% methanol, and stored at − 20 °C. Prior to antibody staining, the specimens were re-hydrated through a decreasing (75%, 50%, 25%) methanol series to PBST (PBS, 0.1% Tween). The specimens were washed in chilled acetone, incubated in acetone for 7 min at − 20 °C, quickly rinsed in double distilled water, washed twice in PBST for 5 min, washed once with PBDT (PBS, 1% BSA, 1% DMSO, 0.5% Triton X-100), and blocked with 5% goat serum (Vector Laboratories, Burlingame, CA) in PBDT. Primary antibody staining (1:10 dilution) was done at 4 °C overnight, followed by three washes with PBDT for 10 min and incubation with goat anti-mouse secondary antibody (1:500) (ThermoFisher, Waltham, MA) at 4 °C overnight. The specimens stained with secondary antibody were washed in PBDT three times for 10 min, cleared in 50% and 75% glycerol, mounted in 75% glycerol, imaged, and photographed. Cardiac looping was also determined at 3, 6, 12, and 19 dpf by visual inspection from the ventral side under a stereomicroscope after specimens were anesthetized with 2 µg/ml MS222 (Tricaine; Western Chemical Inc, Ferndale, CA). Statistical significance of the results was determined by the Chi^2^ test.

### Determination of liver and pancreas asymmetry

The positioning of the liver and pancreas with respect to the midline was determined at 60 h post-fertilization by in situ hybridization with an RT-PCR generated probe for the *cystathionine ß-synthase a* (*cbsa*) gene (Table S1). Previous studies have shown that *cbsa* is strongly expressed in the developing liver and pancreas at this stage of surface fish and cavefish development^33^. In some cases, liver asymmetry was determined by visual inspection from the ventral side as described above. Statistical significance of the results was determined by the Chi^2^ test.

### Survival analysis

Survival analysis was conducted on Mexican surface fish and cavefish larvae separated into groups with D-looped, non-looped, or L-looped hearts. Larvae with different categories of heart looping were isolated in glass bowls in 50 ml of fish system water, fed brine shrimp beginning at 6 dpf, and counted daily. The numbers of surviving embryos were counted under a stereomicroscope after brief anesthetization as described above at the same time every day for the remainder of the experiment. Following counting, the larvae were rinsed several times in fish system water and returned to the bowls. Dead larvae were removed from the bowls daily. Statistical significance was determined using the Cox proportional hazards model in R^54^.

### In situ hybridization

The processing of specimens and procedures for in situ hybridization were carried out as described by Ma et al.^55^. Embryos were dechorionated manually using forceps, fixed in 4% PFA overnight, dehydrated in methanol, and stored at − 20 °C prior to in situ hybridization. The RNA probes used for in situ hybridization were prepared by RT-PCR using oligonucleotide primers (Table S1) designed using sequence information from the *A*.* mexicanus* draft genome^55^. After the completion of hybridization, the embryos were washed with PBST and incubated in BM Purple AP Substrate (Roche, Basel, Switzerland) at room temperature in the dark. After the signal developed, the reaction was terminated by rinsing the embryos in PBST. The embryos were processed through an increasing glycerol series in PBS and photographed using a Zeiss Axioskop compound microscope.

### Pharmacological reduction of nodal signaling

Nodal signaling was inhibited by treatment with SB-431542 or SB-505124 (Sigma-Aldrich, St. Louis, MO), which were dissolved in DMSO to make 10 mM stock solutions. Embryos were dechorionated by treatment with 0.5 mg/ml protease (Protease XIV from *Streptomyces sp*.; Sigma-Aldrich) for 15 s., then rinsed four times in fish system water, and incubated in 1 µM or 5 µM of each inhibitor for 8 h beginning at the shield-75% epiboly stage. Controls were treated for the same period in equivalent concentrations of DMSO. After inhibitor treatment, the embryos were rinsed four times with fish system water, cultured until 3 dpf, and then fixed and processed for MF-20 antibody staining as described above.

### RNA extraction and quantitative real time RT-PCR

Total RNA was extracted using TRI Reagent Solution (Life Technologies, Grand Island NY, USA), treated with RNase-free DNase (Qiagen, Frederick, MD) to remove traces of genomic DNA, and cDNA was synthesized using the SuperScriptTM III First-Strand Synthesis SuperMix Kit and random hexamer primers (ThermoFisher, Rockville, MD). Quantitative real time RT-PCR (qPCR) was done as described by Ma et al.^29,33^ using the oligonucleotide primers shown in Table S2. To confirm the specificity of the primers, BLAST searches were done against the Ensembl Mexican tetra genome database ^34^. Dissociation curves were used to confirm the amplification of single PCR products. The *gapdh* gene was used as the reference gene (Table S2,^−△△Ct^ values, which represent the mean fold change of CF compared to SF mRNA levels. Statistical analysis using ΔCt values was conducted by Student’s t test.

### Sequencing the *lefty2* Gene

The *lft2* exon regions were amplified by RT-PCR from genomic DNA isolated from tail fin clips of individual cavefish males and females using the Phusion High-Fidelity PCR Master Mix (New England Biolabs, Ipswich, MA) and the primers listed in Table S3. PCR conditions were as described by Ma et al.^29,33^. The PCR products were detected by gel electrophoresis, purified with the MinElute PCR Purification Kit (Qiagen, Valencia, CA, USA), and sequenced.

### CRISPR-Cas9 Gene Editing

To edit the *lft2* gene, 50pg of each sgRNA and 300pg Cas9 protein (Cas9 nuclease 2NLS, *S*.* pyrogenes*, Synthego Corp., Redwood City, CA) were co-injected into one-cell stage Mexican surface fish embryos. The two sgRNA were synthesized according to *lft2* sequence information (Ensembl ENSAMXT00000000477): the sequence of the first sgRNA was 5c′-GCUUGCAGCACUGUCAAACA-3′, and the sequence of the second sgRNA was 5′-CAAACAGAAACAUGUGCAUG-3′. Microinjection was carried out as described previously ^33,55^. Injected and un-injected control embryos from the same clutch were cultured at 25 °C. At about 2 dpf, the injected larvae were phenotyped by microscopy, those with axial defects, such as twisted bodies or bent tails, were identified, and some of these larvae were used to extract DNA and genotype the edited sites by nested PCR. For nested PCR, the flanking primers were 5′-GGCTCTAATGTGTCGTGCCT-3′ (forward) and 5′-ACACGATGACAAAACTACCCCT-3′ (reverse), and the nested primers were -5′-TCTAGACGTGATGCAGGGGA-3′ (forward) and 5′-GCCTTAACATACCTATGCCAGC-3′ (reverse). The purified PCR products were assayed for genome targeting efficiency by sequencing. All of the remaining injected larvae and un-injected controls from the same clutch were assayed at 3 dpf for heart looping as described above.

## Supplementary Information

Supplementary Information 1.

Supplementary Information 2.

Supplementary Video.

## Data Availability

Most of the data generated or analyzed during this study are included in this published article (and its Supplementary Information file). Additional datasets generated during and/or analyzed during the current study are available from the corresponding author on reasonable request.
