# Publisher Correction: Maternal control of visceral asymmetry evolution in *Astyanax* cavefish

**DOI:** 10.1038/s41598-021-92148-5

**Published:** 2021-06-15

**Authors:** Li Ma, Mandy Ng, Janet Shi, Aniket V. Gore, Daniel Castranova, Brant M. Weinstein, William R. Jeffery

**Affiliations:** 1 Department of Biology, University of Maryland, College Park, MD 20742 USA; 2 Division of Developmental Biology, Eunice Kennedy Shriver National Institute of Child Health and Human Development, NIH, Bethesda, MD 20892 USA; 3 Present Address: Cave Fish Development and Evolution Research Group, Kunming Institute of Zoology, Chinese Academy of Sciences, Kunming, 650201 Yunnan China

Correction to: *Scientific Reports* https://doi.org/10.1038/s41598-021-89702-6, published online 13 May 2021

The original version of this Article contained errors in the second paragraph of the Introduction.

“The molecular mechanisms of L-R patterning have been extensively studied in traditional vertebrate models^7,8^. An early step in L-R patterning is the leftward beat of cilia in symmetry-breaking organizers, such as the node in mice^9,10^ and Kupffer’s vesicle (KV) in teleosts^11,12^, *pitx2*^13,14,15,16^. The stabilized Nodal-Pitx2/Lefty cascade activates downstream regulatory circuits that control organ morphogenesis on the left or right sides of the body axis^17^. Less is known about L-R patterning events upstream of the symmetry-breaking organizer in vertebrates^18,19^, ^+^/K^+^-ATPase mRNA localization and differences in membrane voltage potentials in *Xenopus*^2^. The influence of maternal factors in controlling L-R shell coiling is well known in snails^20,21^.”

now reads:

“The molecular mechanisms of L-R patterning have been extensively studied in traditional vertebrate models^7,8^. An early step in L-R patterning is the leftward beat of cilia in symmetry-breaking organizers, such as the node in mice^9,10^ and Kupffer’s vesicle (KV) in teleosts^11,12^, which transiently form near the posterior end of the notochord during gastrulation. The leftward directional flow is thought to be sensed by lateral organizer cells, which activate the expression of the TGFß-signaling ligand Nodal and the homeobox gene *pitx2* in lateral plate mesoderm (LPM) on the left side of the axis. The Nodal-Pitx2 signaling cascade then spreads from posterior to anterior in the left LPM and initiates an autoregulatory loop involving the TGFß ligands Lefty1 and Lefty2, which confine the asymmetric signal to the left side of the midline by antagonizing Nodal^13,14,15,16^. The stabilized Nodal-Pitx2/Lefty cascade activates downstream regulatory circuits that control organ morphogenesis on the left or right sides of the body axis^17^. Less is known about L-R patterning events upstream of the symmetry-breaking organizer in vertebrates^18,19^, although the initial patterning steps may be controlled by asymmetries in maternal H^+^/K^+^-ATPase mRNA localization and differences in membrane voltage potentials in *Xenopus*^2^. The influence of maternal factors in controlling L-R shell coiling is well known in snails^20,21^.”

The original Article has been corrected.

