# Supplementary material for: Maternal control of visceral asymmetry evolution in Astyanax cavefish
Source: Sci Rep. 2021 May 13;11:10312. doi: 10.1038/s41598-021-89702-6 (PMC8119719; doi:10.1038/s41598-021-89702-6)
Supplement: Supplementary file 1 — Supplementary Information 1. [file 41598_2021_89702_MOESM1_ESM.docx]

**Maternal Control of Visceral Asymmetry Evolution in *Astyanax* Cavefish**

Li Ma, Mandy Ng, Janet Shi, Aniket V. Gore, Daniel Castranova, Brant M. Weinstein, and William R. Jeffery

**Supplementary Figures and Legends**


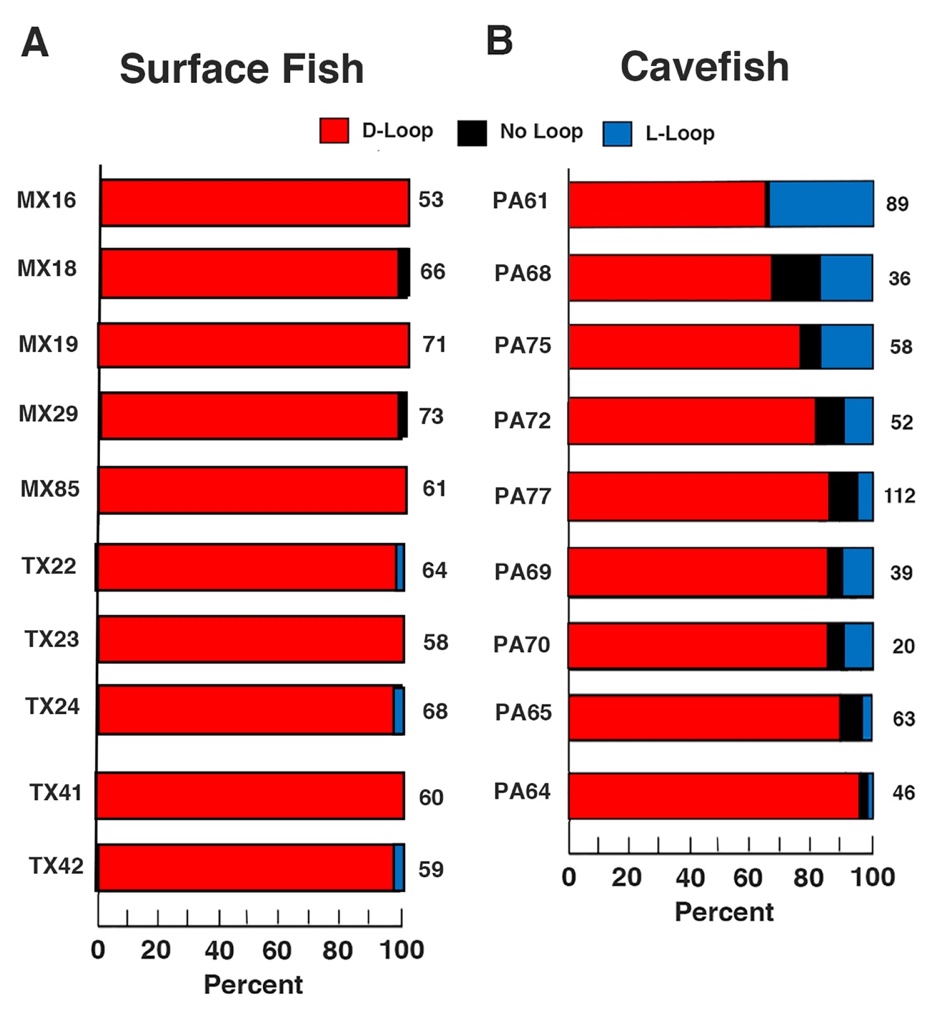


Figure S1. Bar graphs showing heart looping percentages in different surface fish (A) and cavefish (B) families determined by myosin heavy chain antibody staining at 3.5 dpf. Family is indicated on the left side and number of individuals analyzed is shown on the right side of each bar. MXnumber: Mexican surface fish families. TXnumber; Texas surface fish families. PAnumber: Pachón cavefish families.


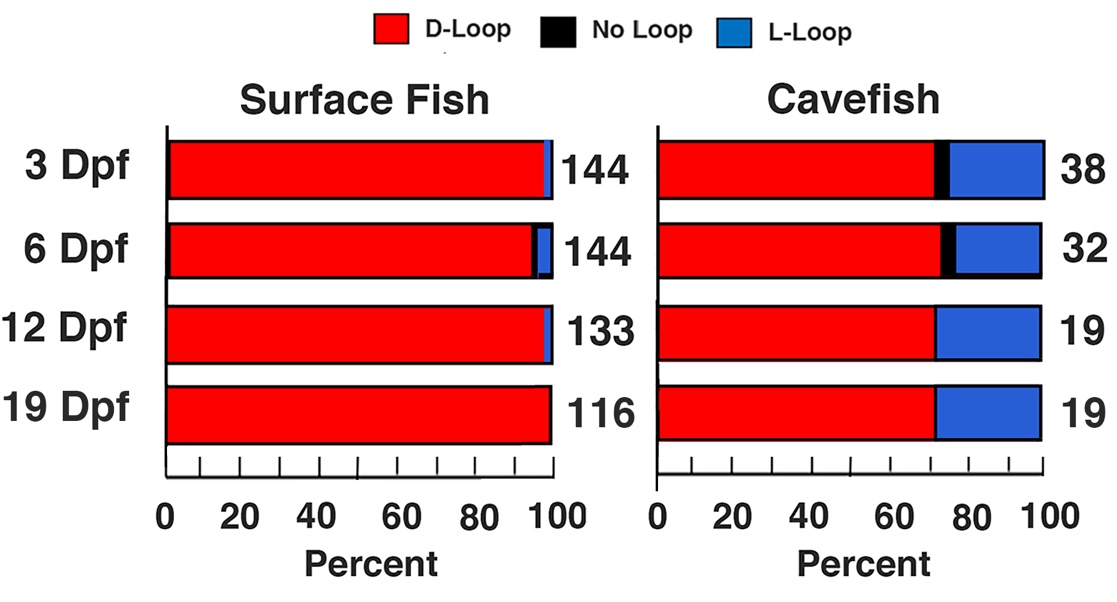


Figure S2. Bar graphs showing the percent heart looping during development in single clutches of surface fish (left) and cavefish (right). Heart looping was determined by visual inspection. Developmental times for both surface fish and cavefish are shown on the left. Number of fish analyzed are shown on the right of each bar. Dpf: Days post-fertilization


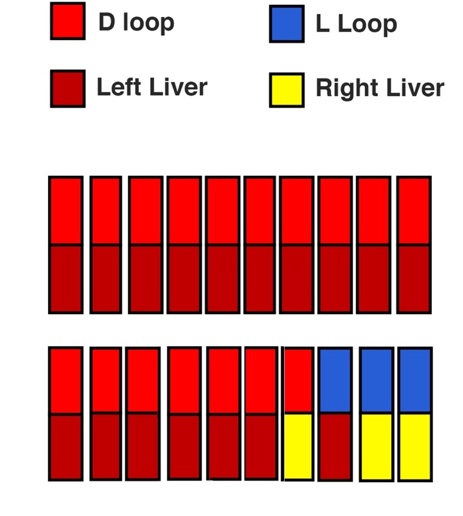


Figure S3. The position of liver development and direction of heart looping compared in 20 individual cavefish. Liver was stained with *cbsa* and heart looping was determined visually and confirmed by myosin heavy chain antibody staining at about 3 day post-fertilization. A total of 16 embryos show D-looped hearts and left livers, 1 embryo shows a D looped heart and right liver, 1 embryo shows an L looped heart and left liver, and 2 embryos show L looped hearts and right livers. Top squares: heart polarity. Bottom squares: liver polarity.


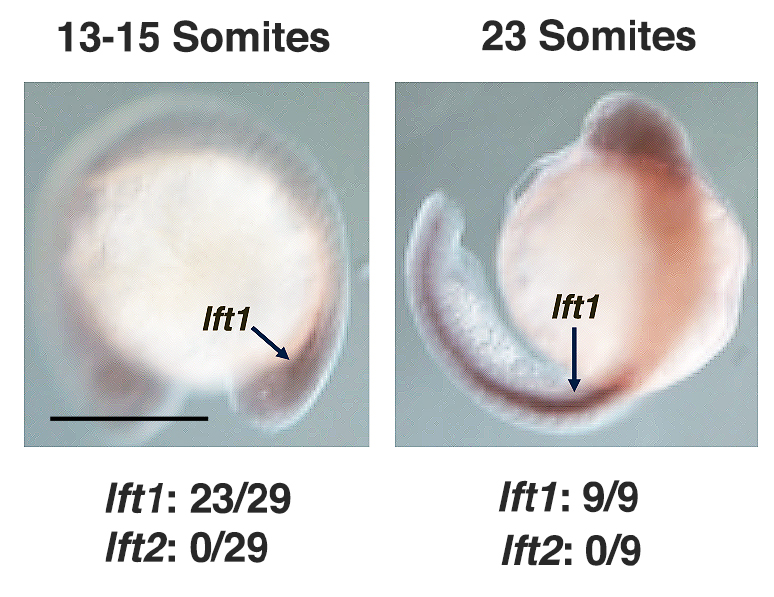


Figure S4. Double *in situ* hybridization of cavefish embryos with *lft1* and *lft2* probes. Stages shown at top of each frame. Number of *lft1* or *lft2* stained individuals per total shown on bottom of frames. Scale bar is 200 µm; magnification is the same in all frames.


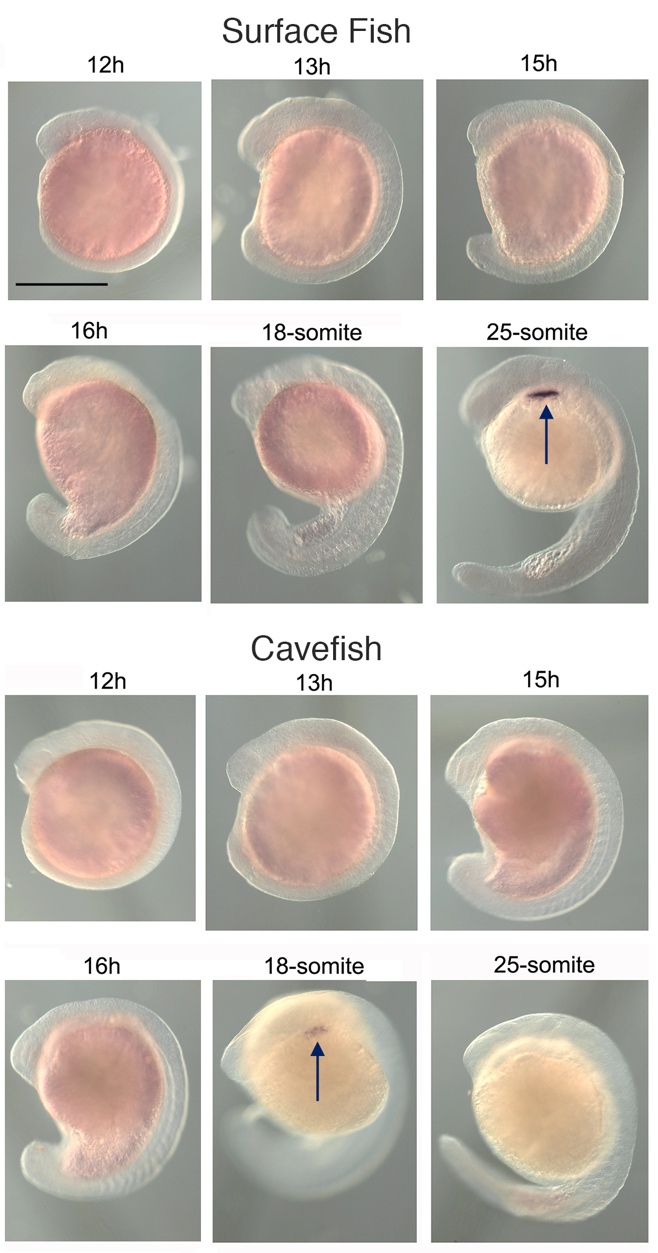


Figure S5. Determination of *lft2* gene expression in left LPM of SF and CF by *in situ* hybridization between the 12 hour (h) and 25-somite stages. The *lft2* gene is expressed strongly in the anterior left LPM at the 25-somite stage in SF embryos (arrow) but weakly in CF embryos at the 18-somite stage (arrow). Scale bar is 200 µm; magnification is the same in all frames.


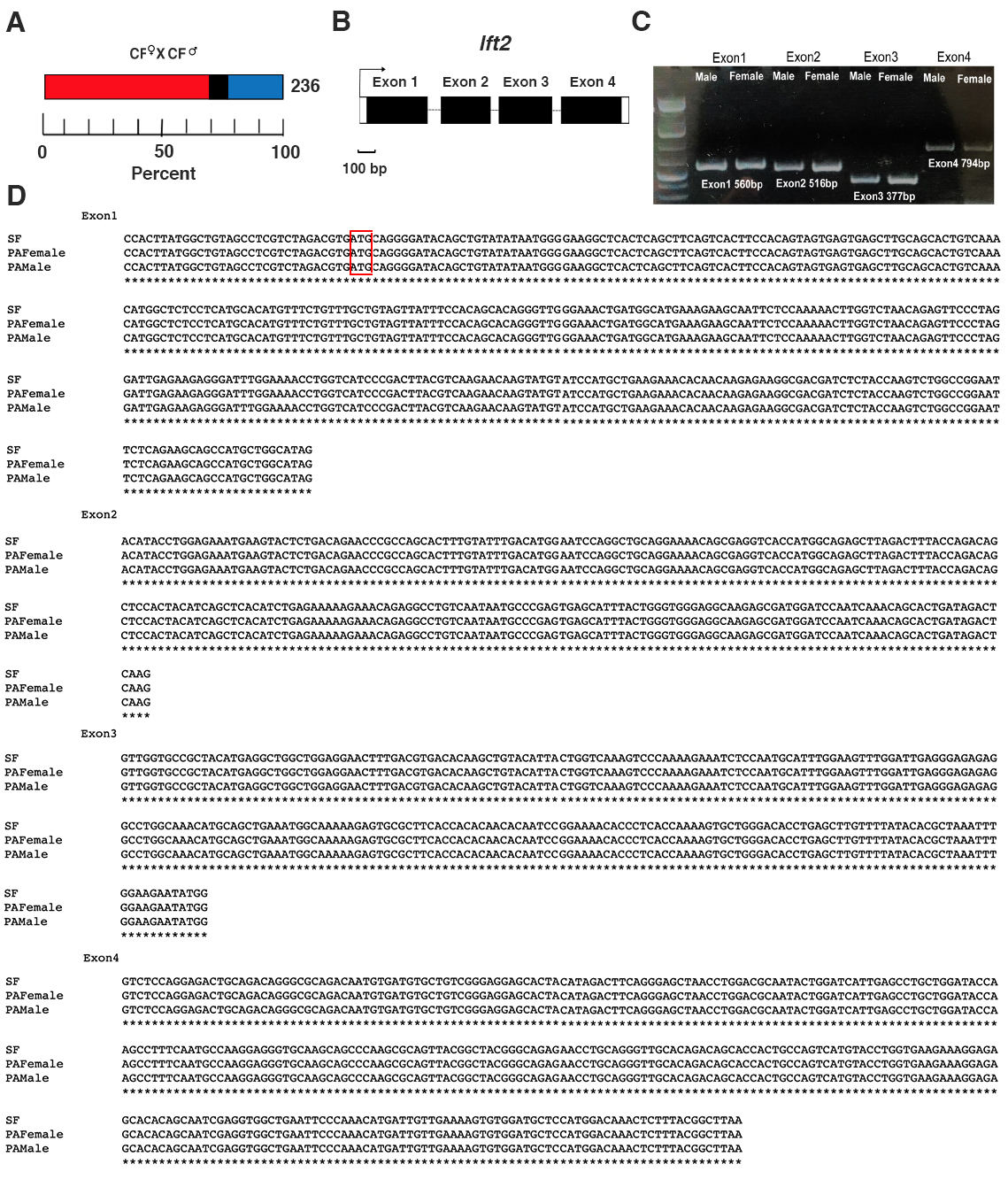


Figure S6. Survey of cavefish genomic DNA for coding region changes in the *lft2* gene. A. A comparison of numbers of progeny with D-looping hearts, non-looping hearts, and L-looping hearts in a cross between the male and female cavefish (CF) parents used for *lft2* sequencing. B. Exon-intron organization of the *lft2* gene. C. PCR amplification of 4 *lft2* exons from male and female cavefish individuals. D. Alignment of *lft2* exon sequences obtained from the male and female cavefish (PA) individuals used in cross in A with the surface fish (SF) *lft2* exon sequence. Red box: ATG translation start site. Asterisks: identical nucleotides.

**Supplementary Tables**

Supplementary Table 1. Oligonucleotide primers used to amplify gene sequences for preparation of RNA probes for *in situ* hybridization.

| Gene | Primers (5’-3’) |
| --- | --- |
| *spaw* | Forward: TTTAACGTGACCGCTCTGCT  Reverse: TGCATGTAGGCGTGATTGGT |
| *lft1* | Forward: CAGGACCCCAGCGATAACTC  Reverse: GCCGCACTTCTCCACTATCA |
| *lft2* | Forward: GGCAAAAAGAGTGCGCTTCA  Reverse: TGTCCATGGAGCATCCACAC |
| *pitx2* | Forward: CCCAAAATGGACGCAAAGGG  Reverse: TATGGTGGCAATTGCAGGGT |
| *cbsa* | Forward: CGCATGCTCATCAGAGACGA  Reverse: GGCAAAGTGATCCGTCTCCA |

Supplementary Table 2. Oligonucleotide primers used in qPCR for preparation of RNA probes.

| Gene | Primers (5’-3’) |
| --- | --- |
| *pitx2* | Forward: CTACACACCCCCTTAGCCAT  Reverse: GTCTTTATCTGCGCACTCGG |
| *lft1* | Forward: GACCCCAGCGATAACTCACT  Reverse: CTGCAGCACTGACCCTGA |
| *lft2* | Forward: GAATCAGTCTTCGCGTTATTTCC  Reverse: GACGTAAGTCGGGATGACCA |
| *ndr1* | Forward: ACCCTAAGCGATACAATGCCT  Reverse: AGCTTCAGAAGACTCTGCATGT |
| *spaw* | Forward: CGCTAAAGACTGTCATCAGGTTG Reverse: AACAACAGCCCGTTTGGTTG |
| *gapdh* | Forward: TCCTGAACTCAATGGCAAGC  Reverse: TTCTCCAAGCGGACAGTCAA |

Supplementary Table 3. Oligonucleotide primers used to amplify *lft2* exons by RT-PCR.

| *lft2* Exon | Primers (5’-3’) |
| --- | --- |
| 1 | Forward: GCACAGTTTGGGCAACAGAG  Reverse: AAAGCAGAGCCTTAACATACCT |
| 2 | Forward: GCATAGGTATGTTAAGGCTCTGC  Reverse: CACGATGACAAAACTACCCCT |
| 3 | Forward: TCAGGGGTAGTTTTGTCATCGT  Reverse: ACACACACCTCAACATTACCTCA |
| 4 | Forward: TGAGGTAATGTTGAGGTGTGTGT  Reverse: AACTGTCGAGTGTTGCCGTA |
