# Supplementary material for: Maternal control of visceral asymmetry evolution in Astyanax cavefish
Source: Sci Rep. 2021 May 13;11:10312. doi: 10.1038/s41598-021-89702-6 (PMC8119719; doi:10.1038/s41598-021-89702-6)
Supplement: Supplementary file 2 — Supplementary Information 2. [file 41598_2021_89702_MOESM2_ESM.docx]

Video Legend

Video showing beating of right (D) looped and left (L) looped hearts in cavefish at 3 days post-fertilization
